# Estimated Acute Effects of Ozone on Mortality in a Rural District of Beijing, China, 2005–2013: A Time-Stratified Case-Crossover Study

**DOI:** 10.3390/ijerph15112460

**Published:** 2018-11-05

**Authors:** Yi Li, Yu Shang, Canjun Zheng, Zhiqiang Ma

**Affiliations:** 1 State Key Laboratory of Severe Weather & Key Laboratory of Atmospheric Chemistry of CMA, Chinese Academy of Meteorological Sciences, Beijing 100081, China; yili@cma.gov.cn; 2 Institute of Environmental Pollution and Health, School of Environmental and Chemical Engineering, Shanghai University, Shanghai 200444, China; yushang@shu.edu.cn; 3 Chinese Center for Disease Control and Prevention, Beijing 102206, China; zhengcj@chinacdc.cn; 4 Institute of Urban Meteorology, China Meteorological Administration, Beijing 100089, China

**Keywords:** time-stratified, case-crossover, O_3_, mortality, seasonal

## Abstract

Studies have shown that ozone (O_3_) has adverse impacts on human health. In China, O_3_ levels have continued to increase since 2010. When compared to the large number of studies concerning the health effects of PM_2.5_ in China, there have been limited explorations of the effects of O_3_. The Beijing region has one of the highest O_3_ concentrations in the country, but there appear to be no published studies regarding the health effects of O_3_ in Beijing. In this study, we applied a time-stratified case-crossover design to explore the effects of O_3_ on cause-specific mortality for a rural location near Beijing over the period 2005–2013. For year-round effects, we found that for all-causes mortality, with a 10-unit increase in O_3_ concentration, the odds ratios (ORs) were in the range of 1.009–1.020 for different lag days. The ORs for cardiovascular mortality with a 10-unit increase in O_3_ concentration were in the range of 1.011–1.017 for different lag days. For warm season effects, the ORs with a 10-unit increase in O_3_ concentration for all-cause mortality were in the range of 1.025–1.031 for different lag days. The ORs for cardiovascular mortality with a 10-unit increase of O_3_ concentration were in the range of 1.020–1.024 for different lag days. Our findings fill a knowledge gap that has hitherto existed in studies regarding O_3_ health impacts, and our results will strengthen the rationale for O_3_ control in China.

## 1. Introduction

In recent years, China has experienced increasing numbers of severe air pollution events. Studies have demonstrated adverse impacts of ozone on human health; among all of the air pollutants, ozone (O_3_) and PM_2.5_ (particles with aerodynamic diameters < 2.5 μm) are believed to have the most significant associations between cause-specific mortality and morbidity, especially cardiorespiratory morbidity [1,2]. Most studies have focused on the adverse effects of PM_2.5_; based on the results of the research, policies regarding PM_2.5_ control have been implemented. In China, strict policies for PM_10_ (particles with aerodynamic diameters < 10.0 μm) and PM_2.5_ control have been implemented. Due to these strategies, concentrations of atmospheric TSP (total suspended particles) and PM_10_ have been decreasing since 1998. Moreover, levels of PM_2.5_ continued to decrease during the period 2010–2016 [3,4,5,6,7,8,9]. However, O_3_ continued to increase after 2010, especially in the three most developed areas in China: the Pearl River delta, Yangtze River delta and Beijing-Tianjin-Hebei [3,4,5,6,7,8,9]. There were no data published concerning O_3_ before 2008. Since PM_2.5_ control policies included reducing both NOx and VOCs (volatile organic compounds), O_3_ concentration increased with a more favorable ratio of NOx to VOCs [10].

Evidence concerning the adverse effects of O_3_ on human health, while being relatively limited, is nonetheless very convincing [11,12,13]. These studies have demonstrated links between short-term O_3_ exposure and adverse health effects, including respiratory illnesses, acute respiratory symptoms, emergency department visits, hospital admissions, and premature mortality. Based on this, the World Health Organization has set O_3_ standards and provided suggestions [14]. In 2012, the Ministry of Environmental Protection of the People’s Republic of China set the following O_3_ air quality standards (GB 3095-2012): class 1 (remote) areas mandate daily 8-h and 1-h maxima of 100 and 160 μg/m^3^, respectively; in class 2 (urban/industrial and surrounding rural) areas, the corresponding values are 160 and 200 μg/m^3^. It is difficult to evaluate the O_3_ standards due to the lack of evidence in regard to the health effects of O_3_ in China. When compared to the large number of studies of PM_2.5_ health effects in China, there have been limited explorations of O_3_. There are only five such studies being reported for Mainland China. Moreover, because of the geographic diversity of the country, these studies are inconsistent in seasonal patterns and health outcomes [15,16,17,18]. Moreover, those studies were all performed in central and southern China, even though the Beijing region has one of the highest O_3_ concentrations in the entire country.

In the present study, we examined the acute effects of O_3_ on mortality in Miyun County, a suburban district of Beijing. Our purpose was to estimate the impacts of O_3_ on human mortality in northern China. To achieve this aim, we applied a time-stratified case-crossover design to explore the lag effects of O_3_ on cause-specific mortality while using a dataset from 2005–2013. We discuss the implications of seasonal modification on the effects of O_3_.

## 2. Materials and Methods

### 2.1. Study Area

**Figure 1:**
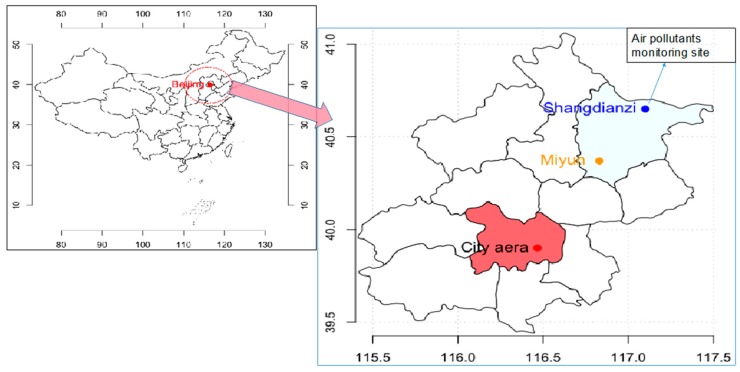
Study area. Red indicates Beijing city districts; light blue indicates Miyun County.

Miyun County is located in the northeast of the Beijing urban area, about 30 km from the center of the city, and is regarded as a background area of Beijing. Air pollution and meteorological data were obtained from the Shangdianzi regional Global Atmosphere Watch (GAW) station (40.39° N, 117.07° E, 293.9 m a.s.l.). This station is located approximately 55 km northeast of the Beijing city area (Figure 1). More details regarding this station can be found elsewhere [19,20].

### 2.2. Data Collection

The datasets consisted of daily mortality records from 1 January 2005 to 31 December 2013. Mortality data were obtained from the Chinese Center for Disease Control and Prevention. We used three death counts, i.e., cardiovascular diseases (ICD-10 code: I00-I99), respiratory diseases (J00-J99), and all-cause mortality (A00-R99).

Daily PM_2.5_ and gaseous pollutants (SO_2_, NOx, and O_3_) were obtained from Shangdianzi station. Shangdianzi station (SDZ, 40°39′ N, 117°7′ E, 293.9 m a.s.l.), is one of the regional Global Atmosphere Watch (GAW) stations in China. The station is located in the northern part of the North China Plain and in the Miyun County of Beijing, approximately 100 km and 55 km northeast of the urban area and the Miyun Township of Beijing, respectively (Figure 1b). Only sparsely populated small villages, and thus insignificant anthropogenic emission sources, lie within 30 km of the site. The station’s instrument building is situated on the south slope of a hill surrounded by mountains in every direction, except the southwest. Due to the valley topography, the prevailing winds at SDZ are from the east-northeast and the west-southwest. Polluted air masses from urban areas and satellite towns of Beijing can therefore be easily transported to SDZ by southwesterly winds, while relatively clean air masses arrive from other wind directions.

Daily meteorological variables (mean and maximum temperature, relative humidity, pressure, wind speed, and direction) were recorded by China Meteorological Administration. From 1 January 2005 to 31 December 2013, 3287 days had data recorded. We used both 8-h maximum ground-level O_3_ and 8-h maximum moving average O_3_.

### 2.3. Statistical Methods

A time-stratified case-crossover design was used to investigate the associations between O_3_ and cause-specific mortality. In this design, “case” days when deaths occurred were compared with control days to assess the effects resulting from differences in exposure to O_3_. Control days were selected to be nearby to case days; in this way, only recent changes in exposure would be compared, and long-term or seasonal variation in exposure could be efficiently eliminated. Conditional logistic regression was used to calculate the odds ratio for cases as compared with controls for a unit increase in O_3_ exposure.

We split our time series data into equally-sized, non-overlapping strata and then used a 35-day stratum length with an exclusion period of three days. The exposure of the case day (index day) was compared with the exposure of the control days, which were matched on the same day of the week within the same stratum. Both single pollutant models and multivariate models (containing all pollutants and meteorological factors) were calculated; separate models were used for all natural cause, and cardiovascular mortality. We also controlled for day of the week (DOW), with Sunday as the reference day. The estimates for O_3_ were scaled to correspond to a 10 ppb increase.

As temperature may have larger effects on mortality than O_3_ and is highly correlated with O_3_, we controlled for temperature by selecting control days within a similar temperature range as the case day.

Odds ratios and 95% confidence intervals (95% CIs) were estimated. The lag structure was an unconstrained distributed lag of the same-day 8-h maximum average ground-level O_3_ concentration (lag 0) and ground-level ozone lag 0-3 days before the case- or control-day. To explore seasonal O_3_ effects on mortality, we divided the data into separate datasets for the warm season (May-October) and the cold season (November-April).

We considered *p* < 0.05 as significant in our statistical tests (all were two-sided). We used R software (version 3.4.3) [21] and the “season” package [22,23] to perform the analysis.

### 2.4. Sensitivity Analysis

We also replaced the 8-h maximum O_3_ concentration with the 8-h moving O_3_ concentration and tested different stratum lengths. The 8-h moving O_3_ concentration means the maximum value of the 8-h moving-average O_3_ concentration. At time T, 8-h moving O_3_ concentration means the mathematical mean value of hour T-7, T-6, T-5, T-4, T-3, T-2, T-1 and time T. Also, we tried different strata length in our model.

## 3. Results

Table 1 shows summary statistics for mortality data, air pollutants, and meteorological factors. The results show considerable variation in O_3_, temperature, relative humidity, and PM_2.5_, i.e., 2.10 to 200.60 μg/m^3^ for O_3_, −15.9 to 32.8 °C for daily mean temperature, 8.0% to 98.0% for relative humidity, and 3.63 to 250.13 μg/m^3^ for PM_2.5_. There were also ranges of 0.07-54.45 μg/m^3^ for SO_2_ and 0.70–90.51 μg/m^3^ for NOx. There were a total of 21,941 all-cause deaths, 1858 respiratory deaths, and 12,275 cardiovascular deaths during the study period.

**Figure 2:**
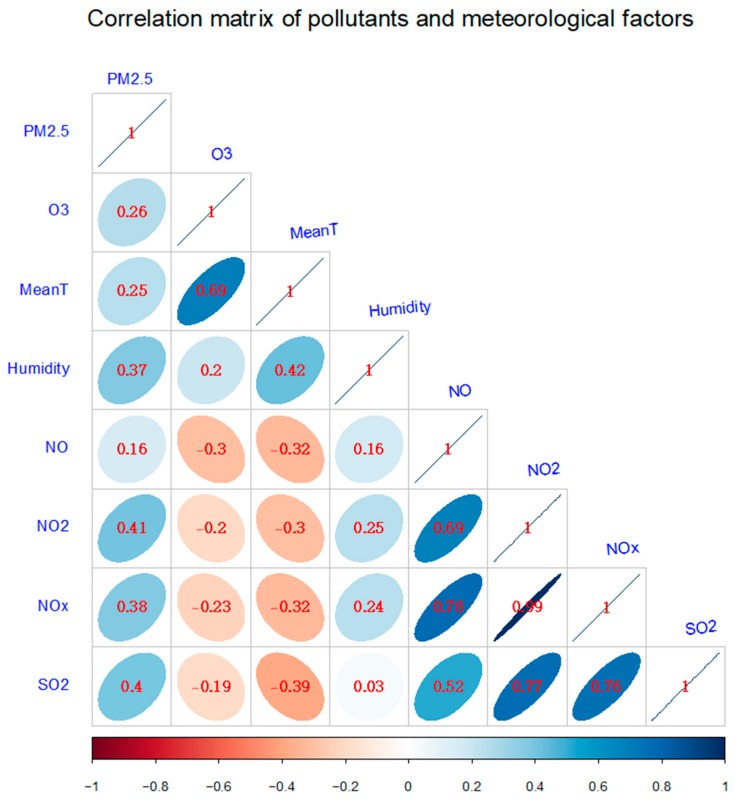
Correlation matrix of pollutants and meteorological factors

**Figure 3:**
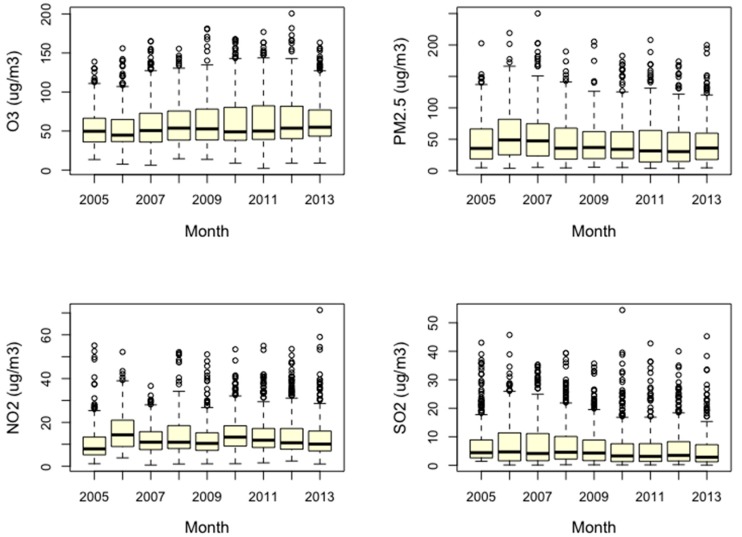
Box plots of air pollutants during the study period.

Figure 2 shows the correlation matrix of air pollutants and temperature. The correlation coefficient between PM_2.5_ and O_3_ was 0.26, much smaller than 0.4, indicating that PM_2.5_ and O_3_ had a weak linear correlation; the two pollutants could be incorporated into a regression model without causing model instability. In addition, there was a strong correlation between temperature and O_3_. The correlation coefficient between NOx and O_3_ was −0.3, and that between SO_2_ and O_3_ was −0.19. Figure 3 presents boxplots for air pollutants during our study period. It could also be seen that the variation of temperature and O_3_ concentrations were relatively larger than those of PM_2.5_, NOx, and SO_2_ concentrations.

### 3.1. Seasonal Characteristics of Health Outcomes, Temperature and Air Pollutants

**Figure 4:**
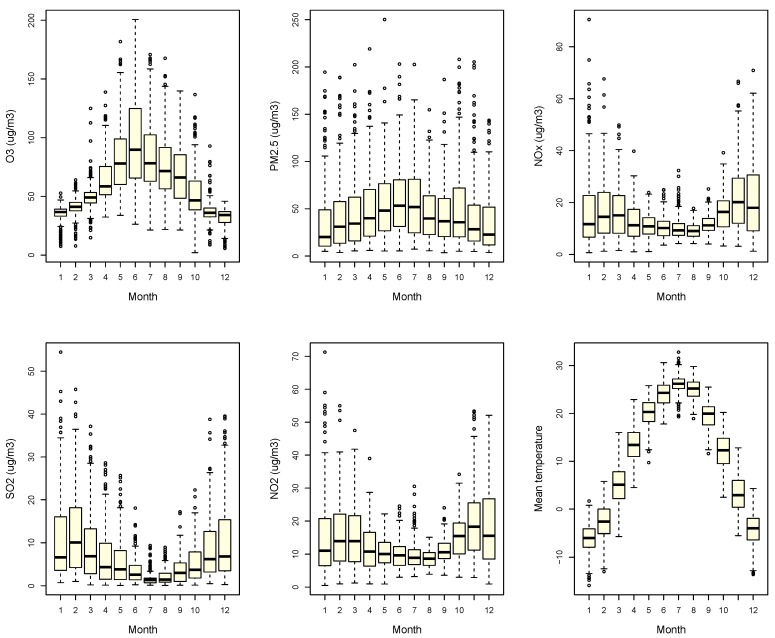
Monthly O_3_, NOx, NO_2_, mean temperature, SO_2_, and PM_2.5_ concentration.

Figure 4 shows that PM_2.5_ concentration did not fluctuate much between the four seasons, but there were significant seasonal variations (*p* < 0.01) in O_3_, NOx, and SO_2_ concentrations. The highest concentration of O_3_ was in summer, followed by spring and fall. The NOx and SO_2_ patterns were opposite, highest in winter, followed by fall and spring.

In our study, the health outcomes also had seasonal patterns due to a complex array of causes, among which temperature, PM_2.5_, and O_3_ were the most important.

### 3.2. Time-Stratified Case-Crossover

Table 2 shows the lag structure of O_3_ effects on mortality throughout one year and during the warm season. A total of 3109 case-days and 53,285 control-days were included in the analysis. Significant (*p* < 0.05) associations between O_3_ and cause-specific mortalities on different lag days were observed, and odds ratios (ORs) increased with O_3_ concentration. Most of the estimates were statistically significant in both single-pollutant and multi-pollutants models, although there were differences between lag days. Estimated effects of O_3_ were moderately reduced but still significant after adjustment for PM_2.5_ and SO_2_ in two-pollutant models. This is probably because, in the atmosphere, O_3_ has a different formation path from those of PM_2.5_ and SO_2_ and thus does not typically covary with these pollutants. The weak correlations observed between O_3_ and these two pollutants indicate that the mortality effect of O_3_ exposure was at least partially independent. In addition, during the warm season, NO_2_ was an important confounder in the association between O_3_ and mortality.

In the single pollutant model (without adjusting for other pollutants) for all-cause mortality for a whole year, O_3_ had significant effects on the current day, lag 1 day and lag 2 day; with a 10-unit increase in ambient O_3_ concentration, the ORs were 1.021 (95% CI: 1.013–1.029), 1.010 (95% CI: 1.002–1.019), and 1.010 (95% CI: 1.001–1.018), respectively. As for cardiovascular mortality, in the single pollutant model, O_3_ had significant effects on the current day, lag 1 day, lag 2 day, and lag 3 day; with a 10-unit increase in ambient O_3_ concentration, the ORs were 1.017 (95% CI: 1.007–1.029) 1.013 (95% CI: 1.002–1.024), 1.011 (95% CI: 1.000–1.022), and 1.012 (95% CI: 1.001–1.023), respectively. There was no significant association observed between O_3_ and respiratory mortality in our study.

We included other pollutants in the two-pollutant models to estimate O_3_ effects. Pearson correlation coefficients between any two pollutants were all < 0.4. Table 2 shows that estimated effects of O_3_ were still significant with slight change after adjustment for PM_2.5_, NO_2_, and SO_2_ for all-cause mortality. However, for cardiovascular mortality, after adjusting for PM_2.5_, the effects for lag 1 day and lag 2 day became insignificant, while those for the current day and lag 2 day were still significant though slightly decreased. After adjusting for SO_2_, O_3_ effects for lag 1 day and lag 3 day became insignificant, while those effects for the current day and lag 2 day were still significant with only slight changes. After adjusting for NO_2_, the associations for different lags became insignificant.

Table 2 also shows all of the significant (*p* < 0.05) effects of O_3_ on health outcomes during the warm season for both single O_3_ models and in the multiple-pollutants model matched for temperature. After matching for temperature to within one degree, we observed significant associations between O_3_ and mortality, and the magnitude of the ORs during the warm season were larger than those for year-round estimates.

The associations between O_3_ and all-cause mortality for every 10-ppb increase in the 8–h maximum O_3_ concentrations were on the current day (OR 1.031, 95% CI 1.005–1.045), lag 1 day (OR 1.028, 95% CI 1.006–1.046), lag 2 day (OR 1.028, 95% CI 1.001–1.041), and lag 3 day (OR 1.025, 95% CI 0.995–1.035). After being adjusted for PM_2.5_, the effects on the current day (OR 1.016, 95% CI 0.999–1.033) and lag 3 day (OR 1.026, 95% CI 1.008–1.044) were still significant. After being adjusted for SO_2_, the effects on current day (OR 1.090, 95% CI 1.037–1.146) and lag 3 day (OR 1.043, 95% CI 0.992–1.106) were still significant. After being adjusted for SO_2_, the effects on the current day (OR 1.090, 95% CI 1.037–1.146) and lag 3 day (OR 1.043, 95% CI 0.992–1.099) were still significant. After being adjusted for NO_2_, all of the associations became insignificant.

The associations between O_3_ and cardiovascular mortality for every 10-ppb increase in the 8-h maximum O_3_ concentrations were on the current day (OR 1.024, 95% CI 1.005–1.045), lag 1 day (OR 1.025, 95% CI 1.006–1.046), lag 2 day (OR 1.020, 95% CI 1.001–1.041), and lag 3 day (OR 1.020, 95% CI 1.002–1.043). After being adjusted for PM_2.5_, the effects on the current day (OR 1.021, 95% CI 1.003–1.053), lag 1 day (OR 1.030, 95% CI 1.003–1.059), and lag 3 day (OR 1.044, 95% CI 1.015–1.075) were still significant. After being adjusted for SO_2_, the effects on the current day (OR 1.078, 95% CI 1.012–1.150) and lag 3 day (OR 1.076, 95% CI 1.010–1.148) were still significant. After being adjusted for NO_2_, all of the associations became insignificant.

### 3.3. Sensitivity Analysis

We replaced 8-h maximum O_3_ concentration with the 8-h moving average maximum O_3_ concentration in all of the models and replaced daily mean temperature with daily maximum temperature and repeated the analysis. The results did not change significantly. We found that the model was robust to changes in stratum length.

## 4. Discussion

We found significant associations between cause-specific mortalities and ambient O_3_ concentration increases. When O_3_ concentration increased, the ORs of all-cause and cardiovascular mortality increased. For both mortalities, the estimated effects of O_3_ were robust with adjustment for other pollutants (PM_2.5_, NO_2_, and SO_2_), while that for respiratory mortality was not, and this is consistent with previous reports [24]. Larger estimates of O_3_ appeared during the warm season for both all-cause and cardiovascular mortality. For year-round effects, the ORs with 10-unit increases of O_3_ concentration for all-cause mortality were in the range of 1.009–1.020 for different lag days before controlling for other pollutants; the range changed to 1.009–1.025 after those controls. The ORs with 10-unit increases in O_3_ concentration for cardiovascular mortality were in the range of 1.011–1.017 for different lag days before controlling for other pollutants; the range changed to 1.010–1.017 after those controls.

During the warm season, the ORs with 10-unit increases in O_3_ concentration for all-cause mortality were in the range of 1.025–1.031 for different lag days before controlling for other pollutants; the range changed to 1.016–1.090 after those controls. The ORs with 10-unit increases of O_3_ concentration in cardiovascular mortality were in the range of 1.020–1.024 for different lag days before controlling for other pollutants; the range changed to 1.021–1.078 after those controls.

Positive ORs estimates for O_3_ in all-cause and cardiovascular mortalities became slightly larger when NO_2_ or SO_2_ were included in the model (Table 2). The reason may be that O_3_ and NO_2_ or SO_2_ are negatively correlated in the atmosphere. This negative correlation had an enhancement effect in the two-pollutant model.

We obtained larger estimates for lag 3 day exposure as compared with those of the current day for both all-cause mortality and cardiovascular mortality during the warm season (Table 2) while controlling for PM_2.5_. These observations are consistent with those from other cities (Shanghai, another metropolitan city in east China) [15] in China. The larger ORs estimated for lag 3 day suggest the accumulation of both acute and less acute health effects over longer periods. The reason why O_3_ could affect the cardiovascular system might be that exposure to O_3_ can induce inflammatory responses. As in vivo and in vitro experiments have demonstrated, O_3_ may mediate a pulmonary inflammatory response; inflammation may subsequently activate hemostatic pathways, impairing vascular function and accelerating atherosclerosis. As cardiovascular mortality accounted for more than 60% of all-cause mortality, a similar result was observed in the estimates of O_3_ on all-cause mortality.

The magnitude of O_3_ estimated effect was much higher than those reported in the USA or Europe. According to a multisite time-series study in the USA, the pooled estimate for 95 urban communities was a 20 μg/m^3^ increase of O_3_ associated with approximately a 0.45–0.60% increase in mortality [12,25]. The concentrations of O_3_ in Miyun County (annual mean 52–65 μg/m^3^ and seasonal mean 35–85 μg/m^3^) were much higher than those in North American cities (14–38 μg/m^3^) [26]. In our smoothed plots of O_3_ concentration against mortality risk, we observed a steeper slope in the high O_3_ concentration range (Supplementary Material, Figure S1). It is worth investigating whether there is any association between long-term O_3_ exposure and the acute effects of O_3_.

Although much higher than those reported in the USA and Europe, the magnitude of estimated O_3_ effect in our study was similar to those of other cities in China [18,27,28]. In four cities in the Pearl River Delta (Guangdong Province, southern China), the strongest effect was on respiratory mortality, and the RR was 1.46~2.61% with a 10 μg/m^3^ increase in O_3_ concentration. In Hong Kong [29], Shanghai [15], and Jiangsu Province [30], the estimated effects (RR) for cardiovascular mortality were 1.31~1.75%. In Wuhan [31], the RR range was 1.03~1.64%. In Zhengzhou [32], the RR range was 1.28~1.79%.

Although the magnitudes of the estimated effects were similar, seasonal patterns were very different between our study and others in China. In our study, the most significant association was observed during the warm season, whereas in the other investigations [30,32], in cities of central-eastern (Zhengzhou, Wuhan, and Yangtze River Delta) and southern (Pearl River Delta and Hong Kong) China, there were significant associations between ambient O_3_ and mortality in the cold season. In the latter studies, after adjusting for PM_10_, the estimated effects of O_3_ on total and cardiovascular mortality increased from September through November, while those for respiratory mortality were only significant from January to August and in December.

The above differences might originate from geographic disparities. When compared to central or southern China, Beijing is a northern city with four distinct seasons as illustrated in Figure 4. Due to those seasons, air pollution in Beijing has a very distinct seasonal pattern. A major impact of seasons is peoples’ exposure patterns to O_3_. In southern China, it is cooler and drier in the “cold” season compared to the “warm” season (higher temperatures and humidity), so people are more likely to open windows or stay outside, increasing their frequency of exposure to O_3_. In the warm season, people are more likely to close windows and use air conditioners. In Beijing, because of lower O_3_ concentrations and lower temperatures in late fall and winter (with heating from 15 November to 15 March), people are exposed to very low levels of O_3_. Moreover, although some significant associations appeared in the single-pollutant O_3_ model, they became insignificant after adjusting for PM_2.5_. Due to the heating supply, a much higher concentration of PM_2.5_ appeared in the cold season; this may “cover” the effects of O_3_. The seasonal pattern in our study is similar to those of northern cities in the USA and Europe [33].

As noted above, we did not observe positive associations between O_3_ and respiratory mortality. The first potential reason is the climate. Jerrett et al. [13] stated in their large cohort study across the USA that it was quite possible that no positive association between O_3_ and respiratory mortality would be found in cool areas (cool in this case meaning a long period of average daily maximum temperature < 25.4 °C). Given Miyun’s cooler climate (yearly mean maximum temperature 17–19 °C) relative to most of the USA, this might be one reason why we found no association between O_3_ and respiratory mortality.

One strength of the present study is that we chose a rural district of Beijing as the study area. Beijing is one of the three highest O_3_ pollution areas (the other two being the Yangtze and Pearl River deltas) in China, but we are unaware of any study regarding O_3_ and health effects in Beijing. It is important to compare the results from various geographic regions in China for policymaking. Moreover, most health effect studies of O_3_ have been conducted in urban areas, and very limited work has been done in suburban and rural areas. As O_3_ and PM_2.5_ are the two air pollutants that have the most significant associations with health effects, and people are often exposed to these two pollutants simultaneously, it is important to evaluate a relatively independent effect of O_3_ on mortality. A rural district as a study object would be a good choice. The O_3_ concentration in the Beijing city area was much lower than that in Miyun County, opposite to the PM_2.5_ concentration. From 2005–2013, the mean concentration of O_3_ in Miyun was 36.0 μg/m^3^, about 1.59 times higher than that in the city area (22.6 μg/m^3^), whereas the mean concentration of PM_2.5_ in Miyun was 44.0 μg/m^3^, only 60% of that in the city area (73.4 μg/m^3^). Since people that were exposed to high concentrations of PM_2.5_ may be more vulnerable to O_3_ pollution, an area with relatively high O_3_ and low PM_2.5_ would be a good choice for exploring the health effects of ozone.

The second advantage is that Miyun County has a cooler summer compared to the city area. The annual daily mean temperature in Miyun was ~1 °C lower than that in the Beijing city area. Moreover, there were only 57 high-temperature (>35 °C) days during 2005–2013 in Miyun, but 102 days in the Beijing city area. Also, there were 626 days with maximum temperature >30 °C in Miyun summers, and 702 d in the Beijing city area. A cooler summer and less developed economy suggest less air conditioner use and more open windows, increasing exposure to ambient O_3_. In our study, we found that the most significant association between O_3_ and health outcomes was in summer. Almost all of the RRs in that season were significantly higher than those in the entire year or other seasons for both all-cause and cardiovascular mortalities. Since summer in Miyun has the least toxic PM_2.5_ and maximum O_3_ concentration, the effects on mortality may be the least confounded.

The third strength of our study is our design for controlling for temperature in the model. As O_3_ correlates to sunlight, the most important confounder is temperature. It is well known that temperature plays an important role in the association between O_3_ and mortality. In our study, the seasonal pattern in mortality was the reverse of that in O_3_: it was the lowest in summer when the highest concentration of O_3_ appeared. This suggested that higher concentrations of O_3_ were associated with lower mortality risks; this illustrated how temperature confounded the association between O_3_ and mortality: higher temperature is associated with lower death risk. It is obvious that temperature had different effects among seasons; the most significant effects appeared in summer and winter with different lag days.

In our study, although stratification was designed to control for the collinearity between O_3_ and temperature, within the strata there still might be a significant correlation between temperature and O_3_ in a city with distinct seasons such as Beijing. In order to control more rigidly for the effects of temperature, we selected control days within a similar temperature range as the case days. An advantage of matching using a confounder is that the shape of the association between the confounder and the dependent variable is not important. This means that the association can take any shape (including non-linear forms), and the estimates would be robust.

One limitation of our study is that compared to the Beijing city area, there is a smaller resident population in Miyun County (470 thousand compared to nine million), and the number of respiratory deaths was relatively small. This small number limited our ability to detect a weak pollution association. Another limitation should be noted in interpreting the results of our study. The exposure data were obtained from only one air pollution monitoring station, and the pollutant measurements may differ from individual exposure levels. Therefore, further investigation is needed to explore this issue. In this case, several factors might be taken into account, such as the correlation between individual exposure or average population exposure and monitoring data. Therefore, the used O_3_ exposure concentration should be most closely related to the individual exposure level when analyzing the health effects of ambient O_3_ exposure.

A third limitation was that we did not include other confounders, such as socio-economic position or individual behavior, which could also influence the health effects of ozone. This was due to data limitation.

## 5. Conclusions

We used a time-stratified case-crossover model to account for the effects of O_3_ on human health, and the analysis provided lag-specific estimates. We conclude that O_3_ had major impacts on cause-specific mortalities in Beijing during the warm season. Our results suggest the need for further investigation of the pathophysiological mechanism of O_3_-associated cardiovascular impact in the northern city. Our work strengthens the evidence for the adverse impact of O_3_ on human health, and our data should be helpful in disease prevention and policy development. Also, our findings fill a knowledge gap that has hitherto existed in studies regarding the health impacts of O_3_. The results will strengthen the rationale for O_3_ control in China.

## Figures and Tables

**Table 1:** Summary of health outcomes, pollutants, and meteorological factors.

	Min	25%	Median	Mean	75%	Max
All-cause mortality	1	5	6	7	9	21
Cardiovascular mortality	1	2	4	4	5	15
Respiratory mortality	1	1	1	1	2	7
PM_2.5_	3.63	18.61	36.89	47.70	66.48	250.13
O_3_(8 h maximum concentration)	2.10	38.10	50.50	59.95	74.85	200.60
Maximum temperature	−9.20	7.20	20.15	18.00	28.50	40.80
Mean temperature	−15.90	0.20	12.90	11.37	22.45	32.80
Relative humidity	8.00	43.00	60.00	58.68	74.00	98.00
SO_2_	0.07	1.62	3.90	6.72	8.91	54.45
NO	0.02	0.36	0.61	1.16	1.15	19.23
NO_2_	0.51	7.54	11.10	13.48	17.17	71.28
NOx	0.70	8.04	11.75	14.64	18.41	90.51

**Table 2:** Odds ratios for daily cause-specific mortality for a 10 μg/m^3^ increase in air pollutants.

		All-Cause Mortality		Cardiovascular Mortality	
		Whole Year	Warm Season	Whole Year	Warm Season
single O_3_	lag0	1.020 (1.013,1.029) **	1.031(1.005,1.045) **	1.017 (1.007,1.029) **	1.024 (1.005,1.045) *
	lag1	1.010 (1.002,1.019) **	1.028 (1.006,1.046) **	1.013 (1.002,1.024) *	1.025 (1.007,1.045) *
	lag2	1.009 (1.001,1.018) *	1.028 (1.001,1.041) **	1.011 (1,1.022) *	1.020 (1.002,1.043) *
	lag3	1.006 (0.999,1.015)	1.025 (0.995,1.035) **	1.011 (1.001,1.023) *	1.015 (0.995,1.035)
	lag4	1.001 (0.993,1.01)	1.006 (0.979,1.019)	0.997 (0.986,1.008)	0.999 (0.979,1.019)
adjusted for PM_2.5_	lag0	1.025 (1.016,1.034) *	1.016 (0.999,1.033) *	1.012 (1,1.025) *	1.021 (1.003,1.053) *
	lag1	1.018(1.009,1.028) **	1.007 (0.989,1.024)	1.008 (0.996,1.021)	1.030 (1.003,1.059) *
	lag2	1.013 (1.004,1.023) **	1.013 (0.996,1.03)	1.010 (0.998,1.023) *	1.019 (0.992,1.048)
	lag3	1.009 (1,1.019) *	1.026 (1.008,1.044) **	1.015 (1.003,1.028) *	1.044 (1.015,1.075) **
	lag4	1.000 (0.992,1.01)	1.007 (0.989,1.024)	0.995 (0.983,1.008)	1.001 (0.973,1.03)
adjusted for SO_2_	lag0	1.017 (1.009,1.026) **	1.090 (1.037,1.146) **	1.017 (1.006,1.029) **	1.078 (1.012,1.15) *
	lag1	1.009 (1.001,1.018) *	1.033 (0.982,1.087)	1.010 (0.999,1.022) *	1.028 (0.965,1.095)
	lag2	1.008 (1,1.017) *	1.032 (0.981,1.086)	1.016 (1.004,1.028) *	1.076 (1.01,1.148) *
	lag3	1.003 (0.995,1.012)	1.043 (0.992,1.099) *	1.010 (0.999,1.022) *	1.000 (0.94,1.063)
	lag4	0.998 (0.99,1.007)	1.050 (0.998,1.106)	0.997 (0.986,1.009)	0.989 (0.926,1.057)
adjusted for NO_2_	lag0	1.021 (1.013,1.03) **	1.01 (0.939,1.087)	1.014 (1.003,1.026) *	1.037 (0.965,1.116)
	lag1	1.014 (1.006,1.023) **	0.962 (0.895,1.035)	1.010 (0.999,1.022) *	0.999 (0.93,1.075)
	lag2	1.015 (1.007,1.024) **	1.01 (0.936,1.091)	1.012 (1.001,1.024) *	0.933 (0.865,1.008)
	lag3	1.007 (0.999,1.016)	0.991 (0.921,1.066)	1.006 (0.995,1.018)	0.955 (0.888,1.028)
	lag4	1.001 (0.993,1.01)	1.033 (0.964,1.108)	0.996 (0.985,1.008)	0.987 (0.921,1.059)

Note: *: *p* < 0.05, **: *p* < 0.01.

## Supplementary Materials

The following are available online at http://www.mdpi.com/1660-4601/15/11/2460/s1, Figure S1: Smoothed plots of relative risk against ozone concentration.
