# Supplementary figures and images for: Estimated Acute Effects of Ozone on Mortality in a Rural District of Beijing, China, 2005–2013: A Time-Stratified Case-Crossover Study
Source: Int J Environ Res Public Health. 2018 Nov 5;15(11):2460. doi: 10.3390/ijerph15112460 (PMC6266742; doi:10.3390/ijerph15112460)

# Smoothed plots of relative risk against ozone concentration

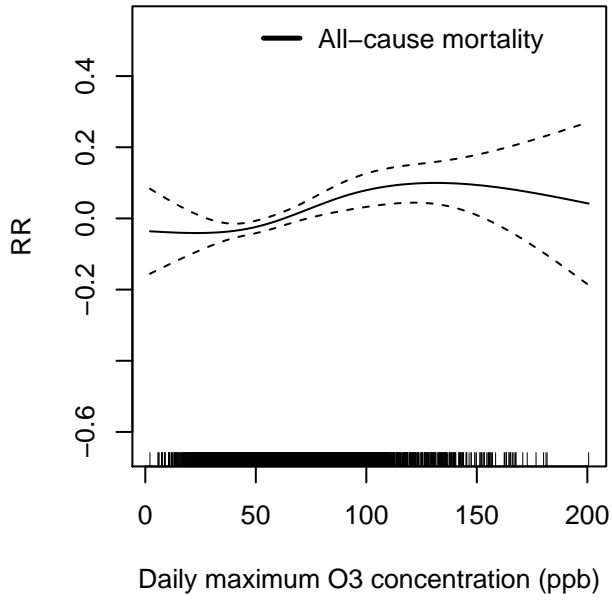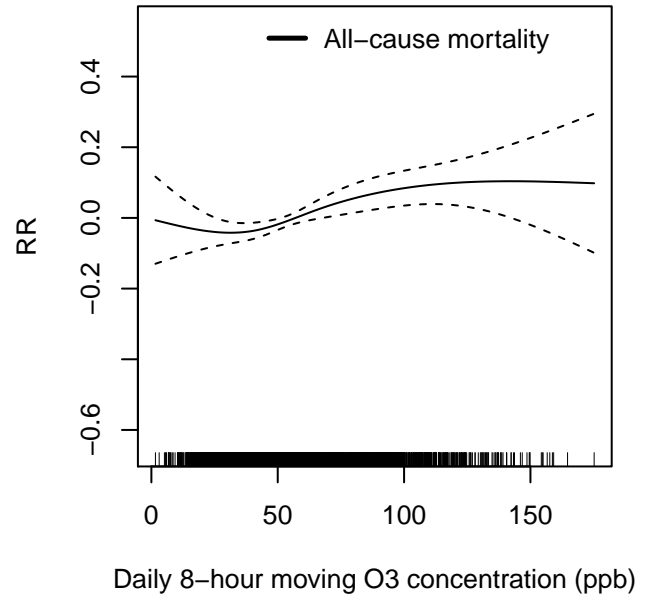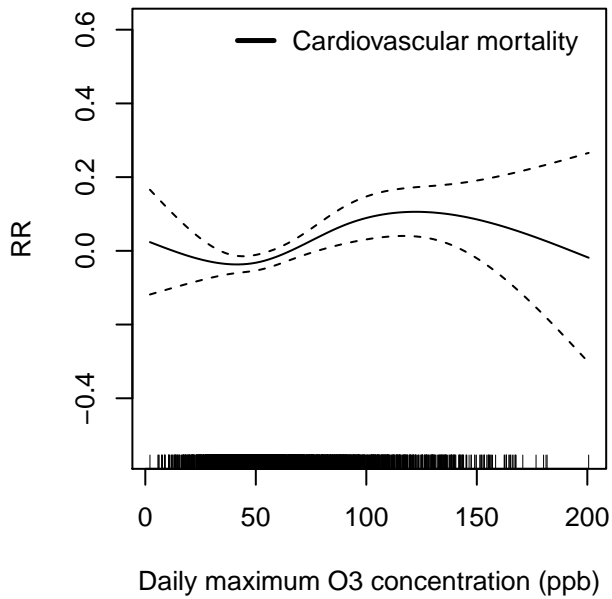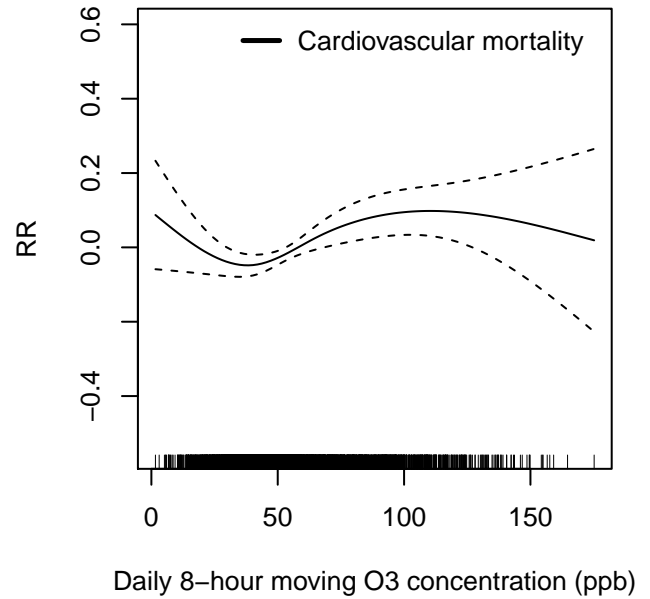

Supplement: Supplementary file 1 [file ijerph-15-02460-s001.zip › suppl..pdf]
